# Healthcare professionals’ perceptions of using a digital patient educational programme as part of cardiac rehabilitation in patients with coronary artery disease – a qualitative study

**DOI:** 10.1186/s12913-023-09997-1

**Published:** 2023-09-21

**Authors:** Jenny Danielsbacka, Caroline Feldthusen, Maria Bäck

**Affiliations:** 1 Department of Occupational Therapy and Physiotherapy, Sahlgrenska University Hospital, Gothenburg, Sweden; 2 Department of Health and Rehabilitation/Physiotherapy, Institute of Neuroscience and Physiology, Sahlgrenska Academy, University of Gothenburg, Gothenburg, Sweden; 3 Institute of Medicine, Department of Molecular and Clinical Medicine, Sahlgrenska Academy, University of Gothenburg, Gothenburg, Sweden SE-405 30

**Keywords:** e-health, Interviews, Myocardial infarction, Patient education, Secondary prevention

## Abstract

**Background:**

Participation in cardiac rehabilitation in patients with coronary artery disease (CAD) remains underutilised. Digital educational programmes, as part of cardiac rehabilitation, are emerging as a means of increasing accessibility, but healthcare professionals’ perceptions of implementing and using these programmes are not known. The aim of the study was therefore to explore healthcare professionals ʼ perceptions and experiences of implementing and using a digital patient educational programme (DPE) as part of cardiac rehabilitation after acute CAD.

**Methods:**

Individual semi-structured interviews were performed with 12 nurses and physiotherapists, ten women with a median age of 49.5 (min 37- max 59) years, with experience of using the DPE as part of a phase II cardiac rehabilitation programme in Region Västra Götaland, Sweden. The interviews were transcribed verbatim and analysed with inductive content analysis according to Graneheim and Lundman.

**Results:**

An overall theme was identified throughout the unit of analysis: “Digital patient education – a complement yet not a replacement”. Within this theme, three main categories were identified: “Finding ways that make implementation work”, “Accessibility to information for confident and involved patients” and “Reaching one another in a digital world”. Each main category contains a number of sub-categories.

**Conclusions:**

This study adds new knowledge on healthcare professionals’ perceptions of a digital patient educational programme as a valuable and accessible alternative to centre-based education programmes as part of cardiac rehabilitation for patients with CAD. The participants highlighted the factors necessary for a successful implementation, such as support through the process and sufficient time from the employer to learn the system and to create new routines in daily practice. Future research is needed to further understand the impact of digital education systems in the secondary prevention of CAD. Ultimately, hybrid models, where the choice of delivery depends on the preferences of the individual patient, would be the optimal model of care for the future.

**Supplementary Information:**

The online version contains supplementary material available at 10.1186/s12913-023-09997-1.

## Introduction

Coronary artery disease (CAD) is the leading cause of death globally [1]. While early mortality after acute CAD has declined in recent years, there is still a great need for the implementation of secondary preventive strategies to improve the long-term prognosis [2, 3]. Secondary prevention provided through comprehensive cardiac rehabilitation programmes is essential to reduce the risk of recurrent cardiovascular events and all-cause mortality and to improve psychosocial well-being [4]. Participation in cardiac rehabilitation has therefore received the highest class of recommendation and level of evidence in European guidelines and should be offered to all patients with CAD [5]. Multidisciplinary cardiac rehabilitation is a complex intervention entailing the optimal use of cardio-protective medication, exercise training, behavioural modification, patient education, and psychosocial counselling [6].

Patient education programmes are designed to allow people with chronic conditions to play an active part in managing their own condition to promote self-care behaviour and risk factor modification with the aim of improving health outcomes [7, 8]. Educational interventions in cardiac rehabilitation have been shown to improve self-reported health behaviours, disease-related knowledge and health-related quality of life and to reduce anxiety and depression in patients with CAD [9–11] but with no clear effects on mortality and hospitalisations [9].

In spite of their established positive effects, centre-based cardiac rehabilitation programmes remain underused in patients with CAD, in Sweden [12] and beyond [13], which limits the effectiveness of the intervention. Barriers to attendance at cardiac rehabilitation are commonly defined as a long distance to hospital, transportation issues and a lack of referral [14, 15]. These challenges reinforce the need to develop alternative modes of cardiac rehabilitation programmes. Digital delivery models are increasingly being suggested as alternatives or adjuncts to centre-based cardiac rehabilitation programmes to increase accessibility, with initial evidence demonstrating similar improvements in physical function, health-related quality of life and cardiovascular risk factors [16, 17]. A systematic review showed that web-based patient educational programmes comprising secondary prevention components had a significant effect on modifiable risk factors and psychosocial outcomes in patients with CAD [18]. Comparisons across studies highlight large variability in digital cardiac rehabilitation programme components, different modes of delivery and various outcomes and, as such, high-quality studies assessing the effects of digital patient educational programmes are still needed [16–18].

The implementation of digital interventions in clinical practice is not without its challenges and depends, among other things, on patients’ perceived usability, utility, and acceptance of the digital system [19, 20]. In addition, external factors, such as the process of system implementation and technology self-efficacy and training, may influence system utility and usability. Health literacy can be defined as “the degree to which individuals are able to access and process basic health information and services and thereby participate in health-related decisions” [21]. Only a few studies so far have aimed to address the digital health literacy skills needed by patients in an e-health context [18, 20]. To be able to describe and identify different barriers and facilitators, the use of a determinant framework may enable the implementation process [22]. Frederix et al. [23] have highlighted potential healthcare professional barriers to digital health deployment, such as infrastructure, incentives, clarity in regulations, knowledge, and training in digital tools. To date, the perceptions of implementing and using digital patient educational programmes as part of cardiac rehabilitation by healthcare professionals are not well known. This is important, as the effectiveness and success of implementing digital interventions in clinical practice will most likely depend on healthcare professionals ʼ attitudes to and perceptions of the digital system.

The aim of the study was therefore to explore healthcare professionals’ perceptions and experiences of implementing and using a digital patient educational programme (DPE) as part of cardiac rehabilitation after acute CAD. An increased understanding of the role of these factors will help in the development of digital cardiac rehabilitation programmes to improve the health and well-being of patients with cardiovascular disease.

## Methods

### Design

This is a qualitative study with individual semi-structured interviews, analysed using inductive qualitative content analysis according to Graneheim and Lundman [24]. One theoretical assumption for qualitative content analysis according to Graneheim and Lundman is that reality can be interpreted in numerous ways and that understanding depends on subjective interpretation [24]. The inductive approach used in the present study means that the analysis is data-driven and characterised by the search for patterns within the text [25]. Another theoretical assumption for qualitative content analysis is made in relation to communication theory, where the theory states that human communication is based on the fact that ‘one cannot communicate’. This means that all texts based on interviews are shaped within interaction between the researcher and participants which could be considered as a communication act [24]. Since the aspect of interpretation always involves multiple meanings the researcher’s interpretation is influenced by pre-understanding. This makes the question of qualifications, training, and experiences within the researcher important [24].

### Participants

The participants were recruited from six sites at five different hospitals that had been part of the implementation process of DPE in Region Västra Götaland in Sweden. Four of the hospitals were county hospitals and one was a university hospital. The inclusion criteria were healthcare professionals who were posted as users of the DPE and who had experience of working with the DPE. The recruitment was executed purposively regarding profession, age, and gender, seeking the broadest possible collection of perspectives according to the chosen method of qualitative content analysis.

### Setting

This study was conducted during phase II cardiac rehabilitation. Patients with CAD in Sweden are offered a centre-based educational programme as a standard component of the comprehensive cardiac rehabilitation programme, usually initiated soon after hospital discharge. Patients receive information about the centre-based educational programme and the DPE from a physiotherapist during their hospital admission and the information is routinely followed-up in the outpatient clinic after a few weeks by a cardiac nurse specialist. The DPE was developed in collaboration with 1177, the Swedish national platform for healthcare information and e-services, as an alternative or adjunct to the usual centre-based educational programme. During the planning phase, the emphasis focused on identifying barriers or facilitators within different parts of the implementation process; the healthcare professionals, the patients, the context where the DPE was going to be implemented and the strategies that should be used to reach out optimally with the new system [22]. The DPE was launched in March 2020 and, during the pandemic when the centre-based educational programme was put on hold, the DPE was the only available alternative. Both programmes cover similar core content targeting the secondary prevention of CAD, including information on the disease and treatment, risk factors, lifestyle habits such as physical activity and exercise, healthy food choices, smoking cessation and alcohol use, medications, and emotional responses. The DPE consists of 13 modules covering texts, illustrations, and short video clips. There are also interactive functionalities with opportunities for patients and healthcare professionals to send messages and for patients to fill in a questionnaire (Alcohol Use Disorders Identification Test, AUDIT) where the results are shared with the healthcare professionals in the DPE. All the information in the DPE is only available in Swedish and taking part in the DPE requires patients to have access to and basic skills in computer and internet use. Patients are also required to have digital identification to log on to the 1177 platform.

### Procedure

The participants were initially contacted via e-mail with written information about the study. A few days later, they were contacted by telephone by author JD. During this conversation, information about the study was given, as well as providing an opportunity for the healthcare professionals to ask questions about the study. Written consent was obtained before participation. Recruitment and interviews were conducted between August 2022 and November 2022.

A semi-structured interview guide, developed by authors MB and JD, consisting of open questions, was used during the interviews to capture different perspectives of perceptions and experiences of both the implementation process as well as working with DPE, all in line with the aim of the study, see Appendix. The interview guide was based both on the researchers´ experiences and on the literature in the field [9, 18, 20–23]. The interviews were conducted by author JD and performed as digital Zoom® meetings and no one else was present. JD presented herself to the participants as a researcher and physiotherapist but with no connection to cardiac care. One pilot interview was performed to test the interview guide. No changes were needed, and the pilot interview was included in the analysis. The interviews were recorded in Zoom® and lasted 30 min as the median (min 25 and max 45 min). No field notes were taken, and no repeat interviews were carried out. The sound files were used as a unit of analysis after verbatim transcription. The transcripts were not returned to participants for comment, correction, or feedback of the findings. The sample size in a qualitative study depends on the quality and richness of the data and it is not possible to propose a specific number of interviews [25, 26]. In this study, all interviews were performed before commencing the analysis process. After completing twelve interviews no new topics appeared in the unit of analysis, hence a decision was made by the authors not to include further participants.

The study was approved by the Swedish Ethical Review Board (registration number 2022–01783-01).

### Data analysis

The results were analysed using qualitative content analysis according to Graneheim and Lundman [24]. The analysis followed the guidelines of Malterud [27] and Consolidated criteria for reporting qualitative research (COREQ): a 32-item checklist for interviews and focus groups [28].

The interviews were listened to and read several times to get a sense of the whole. Meaning units connected to the aim of the study were identified, condensed, and labelled with a code. The codes were then sorted into sub-categories and categories depending on their similarities and differences. Throughout the analysis process, readings of the unit of analysis at different levels were made to maintain a sense of the whole material. Both manifest and latent content were sought for in the unit of analysis. Manifest content is close to data and latent content is abstract with a more interpretive meaning, all the above according to the method of Graneheim and Lundman [24]. Authors JD and MB conducted initial simultaneous analyses of the interviews and discussed the results throughout the analysis process. To triangulate the results and ensure credibility, the preliminary subcategories and categories were discussed with the third author until consensus was reached. Finally, the underlying meaning in the categories was linked together to create a theme. All the authors are physiotherapists and women experienced in the field of qualitative research. MB has a pre-understanding of the subject through clinical experience with the DPE and patient group, while JD and CF have no pre-understanding of either the DPE or the secondary prevention of CAD.

## Results

Twelve participants, ten women and two men, were included in the study. Thirteen eligible persons were asked to participate in the study, and they all agreed to participate. Due to technical problems with Zoom®, one interview was not recorded, leading to the exclusion of one participant. The participants were nurses and physiotherapists working in the field of phase II cardiac rehabilitation. All the participants had experience of using the DPE when it was initiated at their hospital in 2020 or early 2021 in Region Västra Götaland, Sweden. Demographic data for the participants are presented in Table 1.

**Table 1:** Demographic data of the included participants, *N* = 12

Female / Male (n)	10/2
Age, years	49.5 (37–59)
Profession	
Nurses (n)	5
Physiotherapists (n)	7
Professional experience, years	18 (8–30)
Professional experience working in cardiac care, years	12 (5–30)
Time working with DPE, months	24 (14–48)

N or median (min–max), *DPE* Digital patient educational programme

**Fig. 1:**
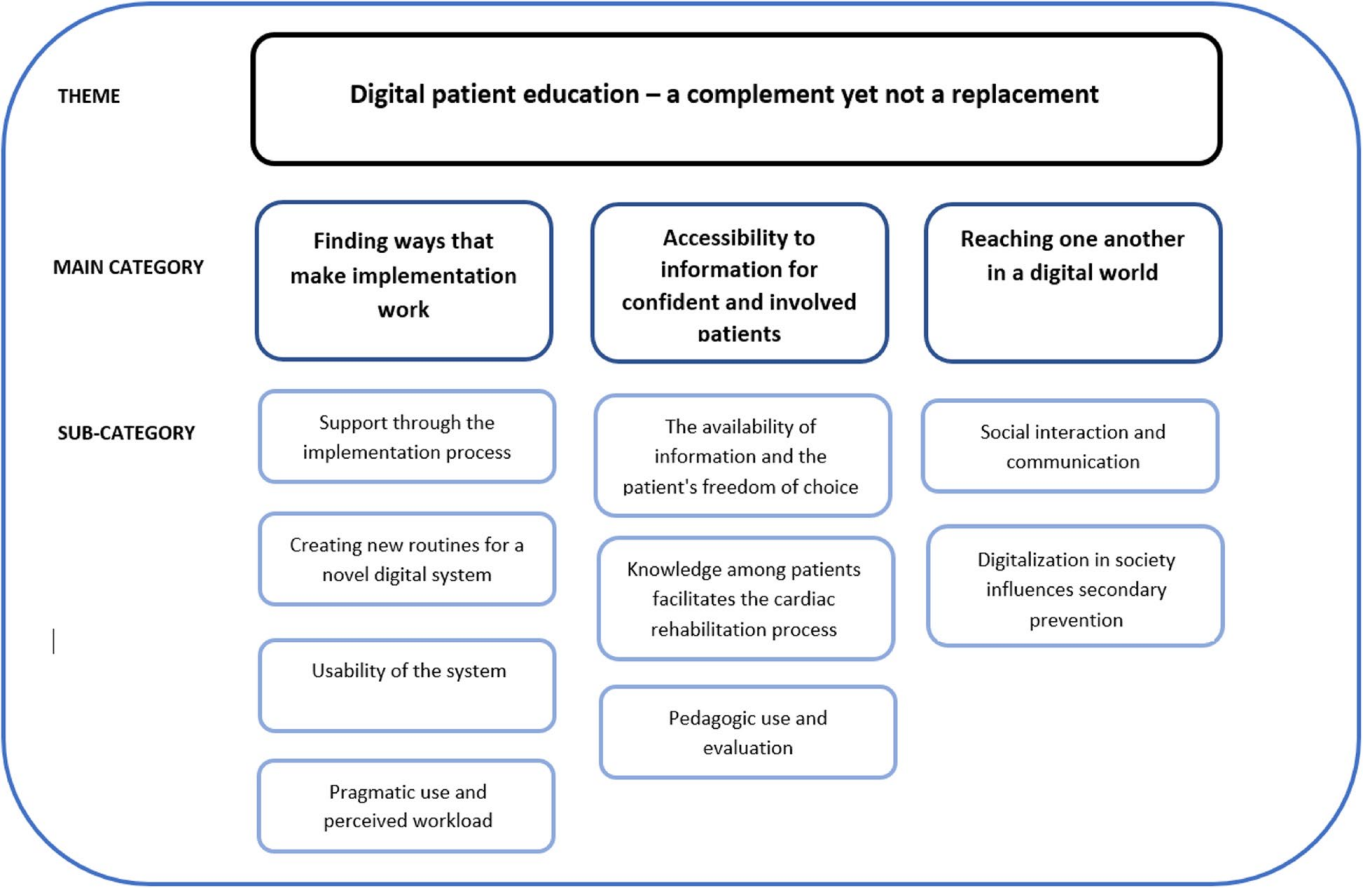
An overview of the overall theme, main categories and sub-categories

An overall theme was identified throughout the unit of analysis: “Digital patient education – a complement yet not a replacement”. Within this theme, three main categories were identified: “Finding ways that makes implementation work”, “Accessibility to information for confident and involved patients” and “Reaching one another in a digital world”. Each main category contains a number of sub-categories, as shown in Fig. 1.

### Digital patient education – a complement yet not a replacement

This overall theme describes perceptions of the DPE being regarded as a complementary source of information, enhancing the opportunity to reach out with information to patients. After taking on all the challenges of an implementation process, it was perceived that giving patients access to both digital and centre-based education was an optimal way of increasing availability to broad-based, well-founded information. Patients obtaining information that enhanced their knowledge of their disease was seen as facilitating visits by giving patients and healthcare professionals common ground to stand on. Among the complementary qualities of the DPE, the advantages of having an up-to-date digital solution that is easy to use were raised as positive, as well as the opportunity to use the DPE as a pedagogic tool. Healthcare professionals said that the opportunity to reach more patients, with the aim of facilitating the lifestyle changes necessary for secondary prevention, could increase by using a DPE. The challenges that were acknowledged were difficulties reaching one another in healthcare through digital solutions.

### Finding ways that make implementation work

In this main category, the participants described taking on the work of an implementation process during a pandemic. Both internal and external facilitators, as well as challenges to this process, were described. Various influencing factors, such as a positive attitude to and motivation towards trying out a new digital patient educational programme, were raised by the participants. This main category comprises the sub-categories: “Support through the implementation process”, “Creating new routines for a novel digital system”, “Usability of the system” and “Pragmatic use and perceived workload”.

#### Support through the implementation process

To be able to work efficiently, the participants described a need for support and guidance of external support when initiating the implementation process. They were content with the introductory information they received that contained both views of the entire DPE system and discussions about the implementation process. The opportunity to receive rapid support when needed was valued, as well as the work manual that was introduced during the process. However, the perception of not needing any support was also raised and it was explained that colleagues helped and learned from one another during the implementation process.

*“It is our work culture that, when we are going to implement something, everyone who works with it should be given as much information as possible. We do it as equally as possible and as efficiently as possible. I can’t concretely say that we had three meetings on Wednesdays at 11, but my experience is that everyone has felt as well informed as possible.”*

*Participant 12*

Additionally, participants perceived that the head of the department could influence the implementation process. Heads with a positive attitude towards the DPE were regarded as encouraging, together with the opportunity to be given time for implementation. However, most participants’ heads of department were not involved practically in the implementation process.

*“We were quite alone, it was really just us… who had to arrange it ourselves… we have our heads and we have things like that, but, no… they probably weren’t that involved in it, we are on our own.”*

*Participant 9*

After having used the programme for some time, the participants expressed a wish for briefings like the ones during the initial implementation process. This was due to new questions arising during the everyday use of the DPE.

#### Creating new routines for a novel digital system

In addition to external support, the participants described a need to solve inner obstacles and to invent new working routines to be able to get the DPE up and running in daily practice. Even though feelings of uncertainty towards and worry about the system were initially present, they said that they became accustomed over time.

*“So when you get the hang of it, I don’t think it’s a problem, but everything has teething trouble, but that’s because we’re not used to it, so, when you get used to it, it’s business as usual.”*

*Participant 5*

During the implementation process, the participants encountered obstacles within the system, but they said that these questions were resolved along the way by the healthcare professionals themselves. The participants also said that not having full knowledge of the system and its functions could be an obstacle to creating new routines, as knowledge needed to be obtained before it was possible to create a solution. Having to create new routines without designated time for it was mentioned as a disadvantage.

*“It was a bit like that at the beginning... it was during a slightly more demanding period, and we didn’t have enough staff down here and you had to cover gaps more or less everywhere, so it was something that we had to do in the middle of… it was a little back and forth.”*

*Participant 1*

#### Usability of the system

An overall perception among the participants was that a digital system should be easy to use. The participants said that the DPE did not require a specialised knowledge of digital systems and IT solutions which enhanced usability.

*“ It’s not that I need to have any special skills, I don’t think so... you don’t need to be an IT hacker for that."*

*Participant 7*

They said that the easiest way to learn to use the system was starting to use it and, through this experience, they learned to work efficiently within it.

*“ I think we got to try it, but then maybe I haven’t absorbed it 100%, but I’ve probably tried and tested my way forward. I’m probably more like that rather than sitting and reading the manual, but it’s probably more of a personality trait, clicking and testing, and, if it doesn’t work, I go to the written description.”*

*Participant 6*

Descriptions of the DPE being easy to use in daily life and the different functions in the system being clear and comprehensible were given. However, perceptions of the system looking old fashioned and being unclear regarding how to find functions and facts about the patients were also mentioned.

#### Pragmatic use and perceived workload

The participants described choosing the pragmatic use of the functions in the system that suited their organisation. Time and resources decided which functions to activate in the DPE. The use of the interactive questionnaires was one example that participants regarded as too time consuming. However, it was felt that secondary prevention could be further improved through the DPE if all the functions in it were utilised optimally.

*“The way I work, I probably work as simply as possible with it, if you like... then, whether it’s right or wrong, I don’t know, but I think it’s better than nothing.”*

*Participant 1*

Descriptions of the workload accumulated by the DPE varied. On the one hand, the DPE was regarded as easy to administer compared with the centre-based educational programme, which required the booking of lecture halls and the co-ordination of the healthcare professionals’ lectures. On the other hand, using yet another system to communicate with patients, as well as other duties associated with it, was described as stressful. During the pandemic, the DPE was seen to facilitate work, as the centre-based education could not be run. However, currently in daily practice, the use of two different educational patient programmes has increased the participants’ workload.

*“It can easily become like that that when there is a new element to be introduced. Now…we´ve had it for a while but still it´s one, one step, an extra step. You still have to log in to another system and add the patient even if it´s not that time-consuming, but it has to be done anyway.”*

*Participant 6*

### Accessibility to information for confident and involved patients

This main category contains perceptions of the impact of using a digital educational programme. Thoughts on what it implies for patients and healthcare professionals but also for secondary prevention after CAD overall were expressed. Using the DPE pedagogically was acknowledged as important in order to be able to increase accessibility to information, as well as learning opportunities for the patients. This category comprises the sub-categories: “The availability of information and the patient’s freedom of choice”, “Knowledge among patients facilitates the cardiac rehabilitation process” and “Pedagogic use and evaluation”.

#### The availability of information and the patient’s freedom of choice

The participants acknowledged the DPE as a way for patients to achieve access to information which could also increase learning about their disease. Having access to the DPE early in the rehabilitation process and for a whole year, the opportunity to repeat information and the flexibility of time and location for use were all acknowledged as facilitators in this area.

*“Availability, you don’t need an appointment, you can choose when, where and how you want to take part in this, it can be on the bus home, so accessibility and freedom of choice both in such practical things and when it comes to time and place, together with the fact that everyone enjoys coming to a physical gathering.”*

*Participant 12*

Moreover, the opportunity for patients to choose the length of each session in the DPE was described as positive.

*“…then there are those who only look through it once and then they think ‘but now I can do this, now I've looked through it’, while some look backwards and forwards, but we are different people when it comes to how much you want to know and how much you think you need to know and so on.”*

*Participant 1*

Access to reviewed information presented in one place was seen to reduce the patients’ need to search for adequate information elsewhere. Perceptions that the patients themselves have a responsibility to enter the DPE to access the information were expressed; healthcare professionals can motivate patients, but it is the patient’s choice to use the resources for information. One disadvantage that was raised was that the DPE is only presented in Swedish, which could lead to inequality for patients not having Swedish as their native language.

#### Knowledge among patients facilitates the cardiac rehabilitation process

The participants´ expressed that patients who had accessed the DPE before a follow-up visit at the cardiac rehabilitation unit could be more involved during the visit. When the patients were well informed by taking part of the domains in the DPE, it gave them and the participants common ground to build on. The perception was also that commonly asked questions had often already been answered though the DPE, leaving room for deeper discussions with the patient and their relatives. The participants perceptions were that a greater degree of knowledge among the patients resulted in the patients being more secure in terms of their diagnosis and possibly more compliant with treatment routines. The feeling was also that patients that had utilised the DPE did not contact healthcare as much as patients without access to the DPE.

*“It’s a health benefit, it’s really a benefit for them to become experts on their own disease, the more knowledge and the safer you are as a patient and know what to do and how you can take preventive action, it’s only positive. It also makes my job easier, of course, so there are a lot of benefits.”*

*Participant 9*

#### Pedagogic use and evaluation

The use of the DPE as a pedagogic tool during visits was acknowledged. By accessing the DPE together with the patient during visits, questions about content or how to use the DPE could be straightened out. However, the participants requested a deeper pedagogic idea of how to use the DPE.

*“Then it’s clear that, if you think that it just happened in the introduction, that we started handing it out and thought “wow, we have this and we can hand it out to the patients”, we didn’t have it, we haven’t had anything pedagogic to explain how we can use it in a pedagogically effective way.”*

*Participant 8*

Evaluation of the programme was considered important to the participants. The need for the continuous revision of the content of the DPE was considered necessary to keep the information correct and up to date.

*“Not to think that it’s an education and it’s fixed and finished, because we don’t do that with the physical one either, it’s also one, still a kind of continuous revision. New research findings, that’s one thing, but also the way society looks in general.”*

*Participant 12*

A pedagogic challenge when presenting the DPE to patients was the participants’ own preconceptions of it versus a centre-based patient education. If patients have thoughts that the DPE is a less worthy alternative than a centre-based resource, these thoughts needed to be restrained when trying to motivate patients to use the DPE. In addition, participants highlighted the importance of comparing the DPE and the centre-based educational programme in terms of patient secondary preventive goal fulfilment.

### Reaching one another in a digital world

This main category raises perceptions of digital communication in the whole of society and how it influences cardiac rehabilitation. Being part of this digitalisation process influences communication, which leads to different challenges in daily practice. Having digital solutions for communication was regarded as both positive and negative. Challenges regarding how to reach as many patients as possible were raised, as well as thoughts of inequality due to differences in access to digital equipment for communication. This category comprises the subcategories: “Social interaction and communication” and “Digitalisation in society influences secondary prevention”.

#### Social interaction and communication

One limitation regarding a digital educational programme perceived by the participants was the lack of social interaction, between both patients and healthcare professionals and patient peers. In centre-based education, the social interaction between patients was perceived to create a feeling of connection and security.

*“But I think that the exchange and seeing that everyone can be affected regardless of how you look, I think many people have felt that, perhaps, that’s the difference. Seeing a physical person and perhaps making contact with someone, it could be that you create social contacts that can strengthen your health in many ways, so perhaps many people miss it, that it’s nice to have it.”*

*Participant 9*

*“There are completely different questions when you are sitting with both the person giving the lecture and also other patients around. So it could be ‘yes, that specific question’ and now I'll come up with another question. There will be a different interaction when you sit, it won’t be, even if you have the opportunity to send in a question, it won’t be the same at all.”*

*Participant 4*

The loss of two-way communication when using the DPE made it more difficult for the participants to intercept misunderstandings and to use their clinical intuition in terms of how the patient was actually feeling. However, they stated that this was possible to intercept on other visits to the hospital during the cardiac rehabilitation process.

*“This thing about reading information and perceiving it in your own way, yes, so that you don’t see how the patient reacts when they read the information.”*

*Participant 2*

The function of answering patient questions using the message function in the DPE was perceived as positive and easy and, if needed, an opportunity to access a follow-up meeting. The need to give clear instructions to patients on which matters to address through the message function was raised. Messages on acute issues could be lost, as the message function was not supervised at all times.

#### Digitalisation in society influences secondary prevention

Being able to offer a digital solution for patient education felt professional and up to date, according to the participants. The perception that cardiac rehabilitation needs to keep up with the digitalisation process in society was raised. Descriptions of patients asking for digital alternatives to be able to access information during centre-based education were given.

*“ So, everything, or a lot of it, must be digitised, but now it is still important to keep up with those developments as well, I think. And many people ask for QR codes and say ‘can I read this somewhere else’ and so on... so it feels like this is something that is very much in keeping with the times.”*

*Participant 1*

However, one concern was present among the participants; having a DPE as the only option for patient education could lead to unequal opportunities for the patients to receive information on secondary prevention. Their perception was that some people, in particular the elderly, did not have the digital skills or equipment to be able to use a DPE. If secondary prevention only consists of digital resources, this could lead to exclusion.

*“ It’s because not everyone is that digital, so the patients don’t, they don’t log in, well, either that they are unable to log in or that they don’t own a computer, or a phone or something and are able to. So that’s it, they need this, and they need material, the technical stuff, to be able to log in and not everyone has it.”*

*Participant 10*

The fact that some patients are not willing to use digital resources due to their suspicion about using their digital identifications was also described.

## Discussion

This qualitative study provides new knowledge of healthcare professionals’ perceptions regarding the implementation process, as well as the use of a digital patient educational programme (DPE) for patients with CAD.

The participants’ overall perception was that the DPE is a valuable complement to centre-based patient education. However, different perspectives of why the DPE should be regarded as an adjunct rather than replacing traditional patient education for these patients were highlighted. The participants expressed a concern that easily accessible information retrieved by using the DPE would not compensate for the loss of social interaction and the two-way communication offered in a centre-based education programme. In a qualitative study on determinants of successful coaching within eHealth, regarding lifestyle changes, Brandt et al. [29] found that it is important to establish a relationship with the patient before commencing the digital coaching. The health care professionals interviewed in the study found it essential to meet the patient before starting the eHealth solution and by using hybrid solutions, with both physical and digital meetings, the goals established were more likely to be fulfilled [29]. In the present study the participants perceived it difficult to assess the patients´ well-being and level of psychological distress without the physical contact. The same difficulty is described previously in a study by Helmark et al. [30], where cardiac nurses should assess patients with implanted cardioverter defibrillators digitally. The importance of designing a digital system to foster effective interactions between patients and healthcare professionals to improve health outcomes has previously been discussed [18]. Different viable tools, such as message functionalities and chat sessions, have been identified for this purpose. There is, however, a need further to elaborate whether social support offered digitally has the same effect on self-management behaviour as traditional educational programmes.

Another challenge that was brought up by the participants was the potentially increased workload for healthcare professionals when offering both digital and centre-based patient educational programmes when cardiac rehabilitation opened up after the Covid-19 pandemic, in terms of taking more time but also the challenge to have more simultaneous tasks. On the other hand, a study by Pena et al. [31] has demonstrated that providing digital patient education after cardiac surgery enhances patient engagement and satisfaction and that patients feel more prepared when coming to out-patient follow-up visits at hospital. This can in turn positively impact healthcare professionals’ workload and increase person-centred care, as it allows the cardiac rehabilitation team to answer questions prepared by patients instead of delivering all the information. This aspect was also highlighted by the participants’ in the current study. However, not all patients that had access to the digital patient education system actually engaged with the platform [31]. This also highlights the fact that, although a large amount of research is being conducted on the evaluation of new digital delivery systems in cardiac rehabilitation, they should not replace traditional centre-based care but instead supplement it [32]. This view is in line with the participants’ perceptions in the current study, as they felt it was important to keep the traditional educational programme, as the DPE on its own was not considered sufficient. This recommendation aligns with the recently published data from the SWEDEHEART annual report, showing that, among the patients that participated in an educational programme after a myocardial infarction in 2022, approximately half the patients chose to attend a DPE [33].

In the present study the participants perceived patients that had taken part of the DPE as more involved and more inclined to ask questions during visits. Health literacy has been noticed as important for patients with cardiac disease [21]. Dunn and Conard [34] have developed The Health Literacy Instructional Model where they point out necessities when working with digital tools and education for patients with cardiac disease. The model includes five domains of health literacy: knowledge, numeracy, navigation, communication, and decision making [34]. Knowledge is a basic prerequisite of being able to take part on decision making regarding one’s disease, and by offering different ways of assimilating knowledge, it is possible to reach different patients [35].

A recently published systematic review revealed that telehealth interventions in the secondary prevention of CAD delivered alone or in combination with centre-based alternatives resulted in favourable changes, including improved smoking status and lipid profile [36]. Although the studies included in this systematic review assessed a broad perspective of core components of cardiac rehabilitation, we hypothesise that a DPE could be offered to patients who are unable to attend centre-based cardiac rehabilitation, or as an adjunct to cardiac rehabilitation, to increase accessibility and flexibility in patient education after CAD. However, more research is still needed to evaluate the patient perspectives and effectiveness of a DPE in the secondary prevention of CAD. Ultimately, hybrid models of patient educational programmes, including both centre-based and digital components, where the choice of delivery depends on the preferences of the individual patient, would be the optimal model of care for the future [32].

As the DPE was launched in the early phase of the Covid-19 pandemic, the temporary shutdown of centre-based cardiac rehabilitation services triggered the rapid, broad-based implementation of the DPE. Even though this process was faster than planned, the participants were dedicated to making the implementation work in order to be able to offer patients some education alternative during the Covid-19 pandemic. During the implementation process, the participants highlighted factors necessary for a successful implementation, such as support through the process and sufficient time from the employer to learn the system and to create new routines in daily practice. These needs are in line with a study by Damschroder et al. [37], which shows that several variables are important during an implementation process in healthcare, i.e. identifying outer and inner settings of the organisations involved in the process, revealing, for example, social, political, economic and cultural settings and contexts.

The willingness to take part actively in the implementation process has been found to be important, as the individuals involved within the organisation have different individual, organisational, cultural, and professional ideas, as well as numerous norms, interests, and individual mindsets [37]. In the present study, all the hospitals were located in one region in Sweden and all the participants were physiotherapists or nurses working in the field of cardiac rehabilitation, which could imply fairly common ground to stand on when the implementation of the DPE was initiated, possibly leading to an easier implementation process.

Lynch et al. [38] have described the implementation process as being divided into three parts: before, during and after implementation, where different questions need to be raised in the different phases to develop the process and its results. After having evaluated the “after part” in the present study, with its rapid implementation process, the organisation around implementation was formalised and structured. For example, a website with manuals, instruction videos and other support was created to facilitate forthcoming implementation processes.

The participants raised concerns that using the DPE could eventually lead to inequality, as not all patients, particularly the elderly, had the opportunity to participate in a digital intervention. However, in a study including more than 300 patients with cardiac disease (mean age of 61.7 ± 14.5 years), Buys et al. [39] showed that 91% of all participants regularly accessed the internet and that different choices of technology-based cardiac rehabilitation appealed to different patients. The older patients in this study preferred web-based options rather than applications [39]. In spite of the potential of digital health systems to support the elderly, the most persistent barrier to digital health adoption is low socioeconomic status [23], which needs to be considered in the further implementation process of the DPE.

Another way to resolve the eventual lack of skills among patients could be to offer patients more education on how to use the DPE. This could be time consuming for the healthcare professionals; however, it could lead to more equal opportunities for patients to participate. The need to educate patients about digital healthcare has also been highlighted as a prerequisite by the European Society of Cardiology [23], as well as prioritising a user-centred approach, taking the needs and preferences of patients into consideration in the development of digital systems. It is also recommended to develop specific training programmes for health care professionals to increase understanding of new service models resulting from digital care. Optimally these programmes should also support application and adoption of new digital health care [23].

This study has both strengths and limitations. In qualitative research, it is important to strive for trustworthiness, achieved through descriptions of the way the credibility, dependability and transferability of the research process and the results were strived for [24, 25]. The credibility of our results is enhanced by the fact that participants were recruited from five of six hospitals participating in the study. Moreover, all the participants that were asked to participate accepted, thereby minimising the risk of selection bias. The variation among the participants regarding, age, work experience and coming from different healthcare professions enhances the opportunity to obtain a rich variation in perceptions and experiences. Only two men were included in the study, but this reflects the gender distribution among cardiac rehabilitation staff at hospitals in Sweden. The analysis of the results was made by all three authors independently and also in collaboration, thereby reaching agreement on the final results. To enhance dependability, a semi-structured interview guide was used. This ensured that all the participants were asked the same questions, even though all the participants had time to elaborate on each question. Furthermore, all the interviews were performed by the same interviewer (JD). The transferability of the results to other populations of healthcare professionals in cardiac rehabilitation has to be judged by the reader. However, the transparent descriptions of the setting, the participants, the analysis process and also the use of direct quotes in the results section enhance transferability to other cardiac rehabilitation settings.

## Conclusions

This study adds new knowledge of healthcare professionals’ perceptions of a digital patient educational programme as a valuable and flexible adjunct to centre-based educational programmes in the secondary prevention of CAD. Important factors for successful implementation were raised and included sufficient support, time to study the system and the creation of new routines. When implementing digital systems in healthcare, it is crucial to identify potential barriers and facilitators and carefully to plan and adjust the implementation process over time. Future research is needed further to understand the impact of digital education systems as part of cardiac rehabilitation programmes.

### Supplementary Information

**Additional file 1.**

**Additional file 2.**

## Data Availability

The interviews analysed in the current study are not publicly available due to identifying patient data should not be shared. Informed consent was not obtained for publication of patient data. Upon reasonable request, deidentified data may be available from the corresponding author.

## Abbreviations

AUDIT: Alcohol Use Disorders Identification Test
CAD: Coronary artery disease
DPI: Digital patient educational programme
