# Supplementary material for: Healthcare professionals’ perceptions of using a digital patient educational programme as part of cardiac rehabilitation in patients with coronary artery disease – a qualitative study
Source: BMC Health Serv Res. 2023 Sep 21;23:1017. doi: 10.1186/s12913-023-09997-1 (PMC10512603; doi:10.1186/s12913-023-09997-1)
Supplement: Supplementary file 1 — Additional file 1. [file 12913_2023_9997_MOESM1_ESM.docx]

Appendix

**Interview guide for the study:**

**“Healthcare professionals ̓ perceptions of using a digital patient educational programme as part of cardiac rehabilitation in patients with coronary artery disease – a qualitative study**”

- Can you tell us about your perceptions and experiences of working with the digital patient educational programme?
- What are your experiences of the implementation process for the digital patient educational programme?
- What are your thoughts on how easy it is to use the digital patient educational programme?
- What are your thoughts on the usefulness of the digital patient educational programme?
- After using it for a while, can you tell us about your attitude to working with the digital patient educational programme?
- Can you tell us how you perceive the benefits of the digital patient educational programme?
- What is your opinion of the importance of the digital patient educational programme for the patients?
- Is there anything else you would like to tell us about?
